# Parental perspectives on youth sport specialization: insights on motivators across specialization levels

**DOI:** 10.3389/fspor.2026.1736749

**Published:** 2026-02-10

**Authors:** Josh Riesenberg, David M. Bazett-Jones, Julie Dyke, Lauren Butler, Gregory A. Walker, Tamara C. Valovich McLeod, Tracy Zaslow, Traci Snedden, Eric Post, Sophia Ulman

**Affiliations:** 1 Movement Science Lab, Scottish Rite for Children, Frisco, TX, United States; 2 Department of Health and Human Performance, High Point University, High Point, NC, United States; 3 The Children’s Hospital of Philadelphia, Philadelphia, PA, United States; 4 Department of Physical Therapy, Florida International University, Miami, FL, United States; 5 Sports Medicine Center, Children’s Hospital Colorado, Aurora, CO, United States; 6 Department of Orthopedics, University of Colorado School of Medicine, Aurora, CO, United States; 7 Department of Athletic Training, A.T. Still University, Mesa, AZ, United States; 8 Department of Orthopedics, Cedars Sinai, Los Angeles, CA, United States; 9 College of Nursing, University of Colorado Anschutz, Aurora, CO, United States; 10 Sports Medicine Research Laboratory, United States Olympic & Paralympic Committee, Colorado Springs, CO, United States; 11 Department of Orthopaedic Surgery, University of Texas Southwestern, Dallas, TX, United States

**Keywords:** activity survey, decision-making, injury prevention, motivating factors, sport participation, sport specialization, youth sports

## Abstract

**Introduction:**

Sport specialization is a decision involving both child and parent. Understanding what drives this decision is crucial given known sport specialization implications which may have negative impacts on an athlete's well-being. This study examined the extent to which various factors motivated parents in their decision-making regarding their child's sport specialization status and to determine whether these motivators varied based on the child's level of specialization.

**Methods:**

279 responses were obtained, and respondents were categorized by their child's specialization status and level. Descriptive statistics were performed.

**Results:**

65% of motivators showed significant differences by specialization status and 30% of motivators varied significantly by level of specialization. Factors like lack of time, fear of injury, and social connections proved to be powerful motivators with group differences. The low specialization group was more likely to disagree that Parent Lacks Time, Parent Fear of Injury, Team/League Rules, and Child Desire for Sport Advancement were motivators as compared to the high specialization group.

**Discussion:**

Despite known risks, the number of specialized youth athletes continues to rise. Educating sport leaders on appropriate sport volumes, or adjustment of schedules to allow for participation in multiple sports, could result in valuable outcomes.

## 1 Introduction

The ability to have fun, be physically active, improve athletic skills, and advance in sports are important factors guiding children and parents in the decision whether to participate in sports (1). However, as the volume of participants and competition level continue to grow in youth sports, the choice of whether to specialize in a single sport remains a widely debated topic. Sport specialization is a complex and increasingly prevalent trend in youth sports. Experts define *sport specialization* as “intentional and focused participation in a single sport for a majority of the year that restricts opportunities for engagement in other sports and activities” (2). However, there is a gap in knowledge behind the decision-making undergirding why a youth athlete would choose to specialize in a single sport.

There are multiple factors that impact the decision to specialize in sport, ranging from the child's intentions to play at an advanced level, recommendations from coaches and personal trainers, as well as resource availability of the family and community. Surveying active youth has revealed specific motivations behind sport specialization, such as college scholarship opportunities or personal interest of a single sport more than others (3, 4). Similarly, collegiate athletes have indicated that personal interest was the most important factor for sport specialization while higher skill level in the chosen sport was second (5). Children are often influenced by parents and coaches as they navigate the sport environment, especially at younger ages (6, 7).

One reason that parents may allow their child to specialize in sports early is to provide more opportunities for sport success in the future. A survey involving parents of early adolescent athletes from a variety of sports reported a moderate belief that focusing on one sport year-round would increase the chance of the child playing in high school (89%) and college (87%) to some extent. Nearly all parents responded that it would increase the chance of improving the player's skills in their primary sport (98%) (8). Parents of highly specialized youth hockey players were also more likely than others to believe that specialization improved their child's chances of making a high school team and earning a college hockey scholarship (9). When parents of early adolescent athletes competing in team sports (e.g., soccer, basketball, baseball/softball) were asked about their level of influence on their child to focus on one sport, nearly 40% indicated that they had little to some influence and over 15% reported having considerable influence on their child to focus on one sport (10). Yet, work investigating the motivations and beliefs of the parents who encourage specialization remains limited.

While skill growth is valuable, specializing in a single sport may not always result in sport success or advancement (11). Notably, DiFiori et al. found that Division 1 collegiate athletes specialized in a single sport significantly later than their aged matched peers whose athletic involvement did not reach the collegiate level (12). Evidence also shows that youth who specialize in a single sport may be at risk of burnout, isolation, and injury (13–18). One prospective study found that youth who were classified as moderately or highly specialized experienced a significantly higher incidence of lower extremity injuries than children classified as low specialization status (19). Future risk of injury increases with increased specialization, such that youth with high specialization had the greatest risk for injury, including overuse injuries (16). A meta-analysis from Post et al. found that highly specialized athletes were significantly more likely to suffer an overuse injury as compared to moderately and low specialization athletes (20).

A consensus definition of specialization has been created using three questions tackling a child's involvement in sports and other activities (16). However, sport specialization is a complex decision that involves a myriad of factors involving both the child and parent (21). Understanding what drives the decision to specialize in a single sport is crucial given the known implications of sport specialization which may have a negative impact on an athlete's physical and mental well-being. The purpose of this study was to examine the extent to which various factors motivated parents in their decision-making regarding their child's sport specialization status and to determine whether these motivators varied based on the child's level of specialization. It was hypothesized that a variety of parental motivators would be strongly associated with their child's specialization status and that these motivators would be modulated by specialization level (low, moderate, high).

## 2 Materials and methods

An anonymous online survey was created to explore the extent to which various factors motivated parents in their decision making regarding their child's sport participation. The custom survey was developed via collaboration between sports medicine practitioners (physical therapists, athletic trainers, and sports medicine physicians) and researchers, all considered sport specialization experts, and study procedures were approved by a regional institutional review board prior to recruitment. Face validity was determined by an expert panel of multidisciplinary sports medicine providers. The survey was shared publicly with the community and snowball recruitment encouraged participating parents to share the survey with other qualifying individuals that they knew personally. Data was collected using Qualtrics survey management software (Seattle, WA, USA).

### 2.1 Participants

The target population for this survey was parents of children between 10 and 19 years of age who were currently active in organized sports or who had been active within the past four years. Given the constant changing landscape in youth sports, the survey was designed to include only youth athletes involved in organized sport over the last four years to capture the most current trends in parental decision making and reduce parent recall bias. Survey responses were collected from November 2022 to August 2023. Individuals that did not speak English or meet stated inclusion criteria were excluded from this study.

### 2.2 Procedures

Demographic information was collected from each parent, including age, sex, race, educational background, household income, and geographic location. Parent responses were then recorded using a five-point Likert scale with response options from 1-*strongly agree* to 5- *strongly disagree* for whether each motivator influenced their decision-making regarding their child's sport participation or specialization. The survey included 23 different motivators covering topics such as resource availability, social interactions for the parent and child, physical abilities of the child, and input from coaches and personal trainers.

While a widely agreed upon definition of specialization has not been long established, researchers have attempted to refine what it means to specialize based on factors like time commitment, exclusivity, and intensity. Therefore, sport specialization status was determined by the research team based on parent responses to three survey questions (16): (1) Does your child play a specific sport for more than 8 months of the year that includes regular organized practices, competitions, and/or other structured training?, (2) Has your child quit other sports to focus on a specific sport?, and (3) Does your child's focused participation in a specific sport limit opportunities or time available for other activities such as participation in other sports, academics, extra-curricular activities, time with friends, and community engagement? “*Yes*” responses corresponded to a 1 and “*No*” to a 0. Responses were then summed, and participants were labeled as either not specialized (0), low specialization (1), moderate specialization (2), or high specialization (3). At the end of the survey, parents were provided with a consensus definition of sport specialization (2) and asked if they believed their child to be specialized (yes/no). If specialization was indicated by the parent, the sport(s) of specialization was collected.

### 2.3 Data analysis

All respondents were categorized by their child's specialization status (i.e., specialized or non-specialized), and subsequently, for the specialized group, by their child's specialization level (low, moderate, or high). Descriptive statistics were calculated for all collected measures, including means and standard deviations for continuous data, medians and interquartile ranges [IQR] for ordinal data (e.g., motivator responses), and frequencies for ordinal and categorical data. Given Shapiro–Wilk tests of normality were significant, non-parametric analyses were conducted. Statistically significant differences in motivator responses were considered clinically significant if median Likert values crossed a full integer value. Mann–Whitney *U*-tests were performed to identify differences in motivator responses by specialization status, and a Fisher's test was performed to determine whether specialization status significantly differed from the parents' indication of whether they considered their child to be specialized. For the specialized group, Kruskal–Wallis tests were conducted to determine whether motivator responses differed by specialization level. When significant effects were found, Mann–Whitney *U*-tests were used for *post hoc* comparisons to reveal which specialization levels differed. Analyses were hypothesis-driven and focused on item-level comparisons to preserve interpretability of individual parental motivators across specialization status and level. Significance was concluded using a significance level (α) of 0.05 and Bonferroni corrections were applied for *post hoc* comparisons using a significance of 0.0167. Small, medium, and large effect sizes were determined as 0.2, 0.5, and 0.8, respectively (22).

## 3 Results

The survey took approximately 10 min to complete, and a brief outline of topics can be viewed in Supplementary Appendix A. A total of 291 parents consented and began the survey and 161 parents with a mean age of 44.31 ± 5.78 years (87.0% white) completed the survey (55% completion rate), resulting in responses for 279 children with a mean age of 13.94 ± 2.66 years. The most common sport of participation was basketball (44.4%). However, the respondents' children were involved in a wide variety of sports (Table 1). A summary of relevant demographic information for parent participants and children are included in Table 1. Based on a consensus definition (2), 35% of parents indicated that they considered their child to be specialized. Soccer and basketball were the most common sports in which parents reported that their children were specialized. Parental responses according the specialization definition (16) are as follows: 155 (55.6%) of survey responses answered “Yes” to whether the child plays a specific sport for more than 8 months of the year that includes regular organized practices, competitions, and/or other structured training. 85 (30.5%) of survey responses answered “Yes” the child quit other sports to focus on a specific sport. 135 (48.4%) of survey responses answered “Yes” that their child's focused participation in a specific sport limits opportunities or time available for other activities such as participation in other sports, academics, extra-curricular activities, time with friends, and community engagement. Additional analysis was done on the 35% of respondents who considered their child to be specialized based on the consensus definition. 89 (90.8%) of survey responses answered “Yes” to whether the child plays a specific sport for more than 8 months of the year that includes regular organized practices, competitions, and/or other structured training. 55 (56.1%) of survey responses answered “Yes” the child quit other sports to focus on a specific sport. 73 (74.5%) of survey responses answered “Yes” that their child's focused participation in a specific sport limits opportunities or time available for other activities such as participation in other sports, academics, extra-curricular activities, time with friends, and community engagement. Interestingly, only a little more than half of parents who self-classified their child as being specialized indicated that their kid has quit a sport for another. These findings highlight that parents may most align the meaning of specialization with the amount of time spend participating in the sport annually. Based on the specialization definition (16), the majority of children were classified as specialized (70.7%), with 26.2% classified as low specialization, 25.1% as moderate specialization, and 19.4% as high specialization, representing a significant under classification of specialization by parents. Thus, a difference was observed between parental responses to child specialization and researcher classification of specialization. Eleven of 23 (48%) motivators showed differences by specialization status (specialized vs. non-specialized) and 26% of motivators varied by level of specialization (low, moderate, or high). All motivator responses and distributions are presented in Table 2. Differences in motivators between the non-specialized group and specialized group are presented in Table 3.

**Table 1:** Demographics of parent participants and included children.

Parent demographics	
Age (*N* = 157)	
Average (SD)	44.31 (5.78)
Sex (*N* = 161)	
Male	57 (35.4%)
Female	104 (64.6%)
Marital Status (*N* = 161)	
Never been married	11 (6.8%)
Living with a partner	2 (1.2%)
Married	131 (81.4%)
Divorced/Separated	14 (8.7%)
Widow	3 (1.9%)
Education (*N* = 161)	
Non-bachelor's degree	32 (19.9%)
Bachelor's degree	52 (32.3%)
Master's degree	48 (29.8%)
Beyond master's degree	28 (17.4%)
Household income (*N* = 159)	
<$100,000	41 (25.8%)
$100,000–150,000	42 (26.4%)
$150,000–200,000	34 (21.4%)
>$200,000	42 (26.4%)
Child demographics	
Age (*N* = 279)	
Average (SD)	13.94 (2.66)
Specialization status^16^ (*N* = 279)	
None	82 (29.4%)
Low	73 (26.2%)
Moderate	70 (25.1%)
High	54 (19.4%)
Specialization definition^16^ (*N* = 279)	
Specific sport ≥8 months/year	155 (55.6%)
Quit other sports	85 (30.5%)
Focused participation limits opportunities	135 (48.4%)
Sport (*N* = 279)	
Basketball	124 (44.4%)
Soccer	82 (29.4%)
Track and Field	75 (26.9%)
Baseball	58 (20.8%)
Football	53 (19.0%)
Volleyball	53 (19.0%)
Cross Country	43 (15.4%)
Softball	39 (14.0%)
Swimming/Diving	31 (11.1%)
Golf	29 (10.4%)
Other	205 (73.5%)

Values represent total (*N*), percent (%). Percentages do not add to 100% given athletes may play multiple sports. Reported sports were categorized as “Other” if they received less than 25 participants reported. Examples include field hockey, rugby, bowling, and weightlifting.

^16^Refers to the previously established definition of specialization as explored by Jayanthi et al. (16).

**Table 2:** Number and percentage of Likert-scale survey responses for parental motivators for sport participation.

Motivator	Strongly agree *n*, %	Somewhat agree *N*, %	Neither agree nor disagree *N*, %	Somewhat disagree *N*, %	Strongly disagree *N*, %
Parent lacks time	29, 10.4	61, 21.9	30, 10.8	73, 26.2	86, 30.8
Child lacks time	28, 10.0	54, 19.4	46, 16.5	74, 26.5	77, 27.6
Financial constraints	15, 5.4	74, 26.5	47, 16.8	59, 21.1	84, 30.1
Community lacks accessibility	19, 6.8	25, 9.0	20, 7.2	36, 12.9	179, 64.2
Team/league rules	9, 3.2	24, 8.6	22, 7.9	67, 24.0	157, 56.3
Coach recommendations	43, 15.4	93, 33.3	60, 21.5	36, 12.9	47, 16.8
Parent desire for college scholarship	43, 15.4	52, 18.6	119, 42.7	21, 7.5	44, 15.8
Improve college application	69, 24.7	113, 40.5	78, 28.0	12, 4.3	7, 2.5
Parent enjoys social opportunity	94, 33.7	101, 36.2	63, 22.6	15, 5.4	6, 2.2
Parent enjoys sport connection	79, 28.3	62, 22.2	88, 31.5	21, 7.5	29, 10.4
Parent has made new friends	69, 24.9	131, 47.3	62, 22.4	7, 2.5	8, 2.9
Parent enjoys parent interaction	76, 27.4	118, 42.6	66, 23.8	10, 3.6	7, 2.5
Parent would miss parent interaction	39, 14.1	89, 32.1	78, 28.2	38, 13.7	33, 11.9
Family support/involvement	127, 45.8	104, 37.5	40, 14.4	4, 1.4	2, 0.7
Parent fear of injury	22, 7.9	74, 26.7	45, 16.2	61, 22.0	75, 27.1
Parent desire for sport advancement	41, 14.8	62, 22.4	112, 40.4	31, 11.2	31, 11.2
Conflicts with child socialization	48, 17.3	112, 40.4	59, 21.3	33, 11.9	25, 9.0
Conflicts with sport advancement	67, 24.2	133, 48.0	58, 20.9	7, 2.5	12, 4.3
Child desire for sport advancement	83, 30.0	67, 24.2	50, 18.1	29, 10.5	48, 17.3
Child lost interest in other sports	55, 19.9	116, 41.9	46, 16.6	27, 9.7	33, 11.9
Child lacks athletic ability	10, 3.6	23, 8.3	44, 15.9	64, 23.2	135, 48.9
Child's injury history	8, 4.3	23, 12.2	43, 22.9	37, 19.7	77, 41.0
Personal trainer recommendations	23, 13.9	64, 38.8	42, 25.5	19, 11.5	17, 10.3

Values represent total (*N*), percent (%). Percentages were computed using question-specific sample size, such that all values for a given question equal 100%. Greyed square indicates the response with the greatest % of respondents.

**Table 3:** Median [IQR], *P*-values, and effect size of each motivator for All parents, parents of Non-specialized children, and parents of children determined to be Any level of sport specialized.

Motivator	ALL	Non-specialized	Specialized	*p*-value	*r*
Parent lacks time	1.0 [1.0]	5.0 [1.3]	3.0 [2.0]	**<0.001**	0.31
Child lacks time	4.0 [3.0]	4.0 [1.0]	3.0 [2.0]	**<0.001**	0.32
Financial constraints	4.0 [3.0]	4.0 [2.0]	3.0 [3.0]	**0.006**	0.16
Community lacks accessibility	5.0 [1.0]	5.0 [1.0]	5.0 [1.0]	0.222	0.07
Team/league rules	5.0 [1.0]	5.0 [1.0]	4.0 [1.0]	**<0.001**	0.23
Coach recommendations	3.0 [2.0]	3.0 [2.0]	2.0 [2.0]	**0.025**	0.13
Parent desire for college scholarship	3.0 [1.0]	3.0 [3.0]	3.0 [1.0]	0.075	0.11
Improve college application	2.0 [1.0]	2.0 [1.0]	2.0 [2.0]	0.583	0.03
Parent enjoys social opportunity	2.0 [2.0]	2.0 [2.0]	2.0 [2.0]	0.865	0.01
Parent enjoys sport connection	2.0 [2.0]	2.0 [2.0]	3.0 [2.0]	0.082	0.10
Parent has made new friends	2.0 [2.0]	2.0 [1.0]	2.0 [1.0]	0.056	0.11
Parent enjoys parent interaction	2.0 [2.0]	2.0 [2.0]	2.0 [2.0]	0.858	0.01
Parent would miss parent interaction	3.0 [2.0]	3.0 [2.0]	3.0 [1.0]	0.140	0.09
Family support/involvement	2.0 [1.0]	2.0 [1.0]	2.0 [1.0]	0.279	0.07
Parent fear of injury	3.0 [3.0]	4.0 [3.0]	3.0 [2.0]	**0.005**	0.17
Parent desire for sport advancement	3.0 [1.0]	3.0 [2.0]	3.0 [1.0]	0.084	0.10
Conflicts with child socialization	2.0 [1.0]	3.0 [2.0]	2.0 [1.0]	**0.011**	0.15
Conflicts with sport advancement	2.0 [1.0]	2.0 [1.0]	2.0 [1.5]	**0.015**	0.15
Child desire for sport advancement	2.0 [3.0]	3.0 [3.0]	2.0 [2.0]	**0.012**	0.15
Child lost interest in other sports	2.0 [1.0]	3.0 [2.0]	2.0 [1.5]	**<0.001**	0.23
Child lacks athletic ability	4.0 [2.0]	5.0 [1.0]	4.0 [2.0]	0.387	0.05
Child's injury history	4.0 [2.0]	4.5 [1.8]	4.0 [2.0]	**0** **.** **024**	0.17
Personal trainer recommendations	2.0 [1.0]	2.0 [2.0]	2.0 [1.0]	0.381	0.07

Median [IQR] values are represented as 1 = strongly agree and 5 = strongly disagree. Bold values indicate a significant difference (*p*-value of α = 0.05) between Non-Specialized and Specialized groups.

**Figure 1:**
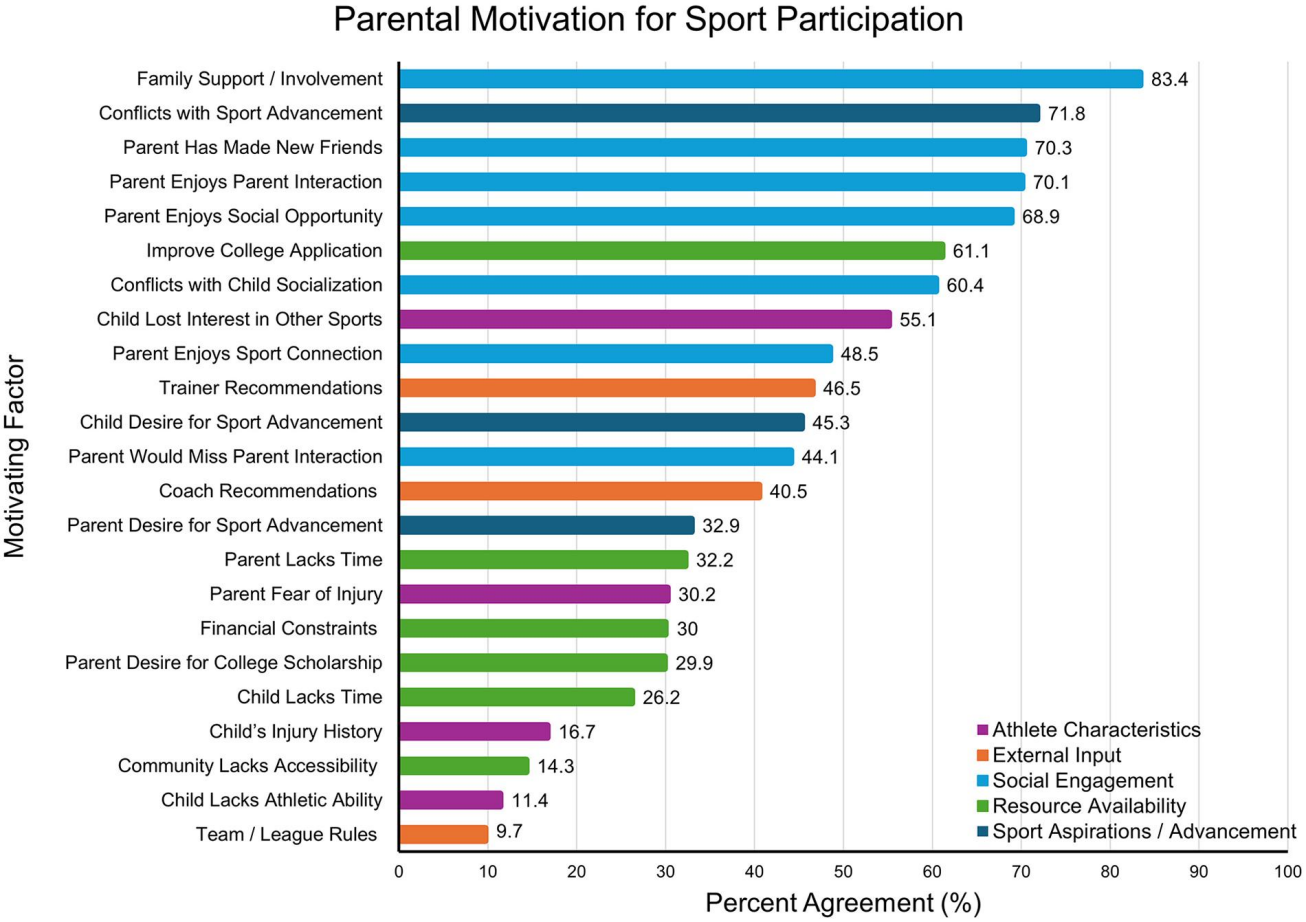
Percent agreement of parental motivation for sport participation. “*Strongly Agree*” and “*Somewhat Agree*” responses were combined to total percent agreement (%).

Family Support/Involvement was the motivator with the highest total percentage of *strongly agree* responses (45.5%). In contrast, Community Lacking Accessibility (64.2%), Team/League Rules (56.3%), and Child Lacking Athletic Ability (48.4%) garnered the strongest disagreement. Parent Desire for College Scholarship (42.7%) and Parent Desire for Sport Advancement (40.1%) both elicited the most neutral responses, indicating that they neither agreed nor disagreed. Lastly, parent-specific social factors, including Parent Enjoys Social Opportunity, Parent Enjoys Sport Connection, Parent Made New Friends, Parent Enjoys Parent Interaction, and Parent Would Miss Parent Interaction all leaned heavily toward agreement (45.9–71.7%) and received little disagreement (5.4–25.4%). A visual highlighting the agreement of each motivator can be found in Figure 1. “Lack of time” variables represent having less time available for allowing of participation in multiple sports.

Parents of a child without specialization (non-specialized) were more likely to disagree that Parent Lacks Time and Child Lacks Time were motivators for participation as compared to parents of a specialized child. Notable differences were observed in non-parent attributes, with the non-specialized group more likely to agree that Conflicts with Child Socialization, Child Desire for Sport Advancement and Child Lost Interest in Other Sports were motivators as compared to the specialized group. Additionally, the non-specialized group was more likely to disagree that Financial Constraints, Team/League Rules, Coach Recommendations, and Parent Fear of Injury were motivators as compared to the specialized group.

Differences in motivators among specialization groups are presented in Table 4. Across specialization levels, the low specialization group was more likely to disagree that Parent Lacks Time, Parent Fear of Injury, and Team/League Rules, were motivators as compared to the high specialization group. The low specialization group was more likely to disagree that Child Lacks Time was a motivator as compared to both the moderate and high specialization groups.

**Table 4:** Median [IQR] of each motivator for parents of children determined to exhibit Low, moderate, and high sport specialization.

Motivator	Low specialization	Moderate specialization	High specialization
Parent lacks time	4.0 [3.0]	3.0 [3.0]	2.0 [2.0]^**L**^
Child lacks time	4.0 [2.0]	3.0 [2.0]^**L**^	2.0 [2.0]^**L**^
Financial constraints	4.0 [3.0]	3.0 [3.0]	3.0 [3.0]
Community lacks accessibility	5.0 [2.0]	5.0 [1.0]	5.0 [1.0]
Team/league rules	5.0 [1.0]	4.0 [1.0]	4.0 [3.0]^**L**^
Coach recommendations	2.0 [2.0]	2.0 [1.0]	3.0 [2.0]
Parent desire for college scholarship	3.0 [1.5]	3.0 [1.0]	3.0 [1.3]
Improve college application	2.0 [1.0]	2.0 [2.0]	2.0 [2.0]
Parent enjoys social opportunity	2.0 [2.0]	2.0 [2.0]	2.0 [2.0]
Parent enjoys sport connection	2.0 [2.0]	3.0 [2.0]	3.0 [1.3]
Parent has made new friends	2.0 [1.0]	2.0 [1.0]	2.0 [2.0]
Parent enjoys parent interaction	2.0 [1.5]	2.0 [2.0]	2.0 [2.0]
Parent would miss parent interaction	3.0 [1.0]	3.0 [1.0]	2.0 [1.0]
Family support/involvement	2.0 [1.0]	1.0 [1.0]	1.0 [1.0]
Parent fear of injury	4.0 [3.0]	3.0 [2.0]	2.5 [2.0]^**L**^
Parent desire for sport advancement	3.0 [1.0]	3.0 [1.0]	2.5 [1.3]
Conflicts with child socialization	2.0 [1.0]	2.0 [1.0]	2.0 [0.0]
Conflicts with sport advancement	2.0 [1.0]	2.0 [1.0]^**L**^	2.0 [1.3]^**L**^
Child desire for sport advancement	3.0 [3.0]	2.0 [2.0]	2.0 [2.0]
Child lost interest in other sports	2.0 [2.0]	2.0 [1.3]^**L**^	2.0 [1.0]^**L**^
Child lacks athletic ability	4.0 [2.0]	4.0 [2.0]	5.0 [1.3]
Child's injury history	4.0 [2.0]	4.0 [3.0]	3.5 [2.0]
Personal trainer recommendations	2.5 [1.0]	2.0 [1.0]	2.0 [1.0]

Median [IQR] values are represented as 1 = strongly agree and 5 = strongly disagree. *Post hoc* results (*α* = 0.167) indicated with an ^**L**^ for significant differences from low and an ^**M**^ for significant differences from moderate.

## 4 Discussion

The purpose of the current study was to determine the influence of motivators for youth sport specialization, as rated by parents, and how motivators differed by specialization status of the youth athlete. It was hypothesized that a variety of parental motivators would differ by child's specialization status, which was supported by the findings of the current study. Most parental motivators showed differences between specialization statuses. It was also hypothesized that parental motivators would vary by the child's specialization level, which was partially supported as nearly a third of motivators differed across specialization levels.

There was strong disagreement that lack of time for both the child and parent, financial constraints, and lack of community accessibility were motivators for parents. This was an unexpected finding as resource availability, or a lack thereof, has often been associated with specialization (23, 24). However, the non-specialized group was more likely to disagree that time and money were motivators for their child's specialization. Parents whose children were classified as highly specialized were more likely to agree that lack of time for both the child and parent were motivators as compared to parents whose child was classified in the low specialization group, suggesting that specialization may occur as a result of limited time. External pursuits, like jobs or volunteering in addition to concurrently demanding sport schedules, may limit multi-sport participation time, resulting in a single-sport focus. The fundamental role of money and time has been observed in previous work; however, prior findings suggest that resource demands were a barrier to participation and specialization (25). Over 75% of baseball parents indicated that the time demands of non-sport activities limited the child's ability to play baseball and a majority stated that the time and cost demands of baseball itself were barriers to baseball participation (23). Whether one sport or many, previous work alongside the present study further reiterate the valuable role of time in youth sport participation.

More than 35% of parents in the present study considered their child to be specialized in sport(s) based on the consensus definition that they were provided. However, 70.7% of children met specialization status per that same consensus definition when assessed by the researchers (16). This discrepancy highlights a consistent and significant disconnect in the understanding of sport specialization between parents and experts, as well as a gap in how we attempt to measure it. Even when provided with a consensus definition, parents may be inclined or influenced to answer in a certain way based on their own knowledge of their child, the additional environmental factors not captured by most specialization questionnaires, and/or the motivators behind the decision to specialize or not. Considering parents were only presented with the figure and not fully educated on the definition, parents may also interpret words or descriptions differently. 44.5% of children were classified as having either moderate or high specialization, a closer representation to the 30% specialization value as determined by parents, highlighting a disparity in the potential overclassification of a three-item scale and the difficulty in identifying low specialization. 84.7% of those who self-classified as being specialized were also grouped as having either moderate or high specialization according to the previously published definition. Future parental education should more clearly define what it means to be a specialized athlete and express the benefits and drawbacks of early sport specialization. While there is some debate in the academic community about what constitutes specialization, more robust scales have recently been published and should be further investigated in future research (26).

Parents of children in the specialized group were more likely to agree that their child's desire for sport advancement and their child's loss of interest in other sports were motivators for specialization, and this was most strongly reported in parents of children with higher specialization levels. The decision to specialize may be attributed to the child's desires rather than the parent's. It is possible that parental influence is lessened as the adolescent enters into their later teens; however, this was not evaluated. A child who is determined to progress in competition, and certainly if they no longer enjoy other sports, may end up quitting activities at the expense of their favorite or best sport (27). Understanding these dynamics will likely require qualitative or mixed-methods studies designed to understand child and parent motivators more clearly.

Parents of children in the specialized group were more likely to agree that fear of injury is related to an increase in the child's current sport participation/training volume or participation in certain sports motivated sports participation choices compared to parents of children in the non-specialized group. Within the specialized group, parents of children classified as highly specialization were also more likely to agree with this sentiment than parents of children in the low specialization group. Parents may purposefully, or unintentionally, drive sport specialization to avoid injury, potentially due to participating in multiple sports, and thus, ultimately reducing the child's total exposures. However, it is also notable that most parents disagreed that their child's injury history motivated which sports a parent allowed a child to participate in. Previous research has reported that over 90% of youth sport parents voiced that specialization would increase chances of getting hurt (23, 24). However, research consistently demonstrates that specialized athletes are at a greater risk for injury (15, 16, 19). Despite the evidence for increased injury risk with specialization, many children continue to specialize. A lack of parental understanding of the known injury risks of specialization may explain this disconnect. Knowledgeable researchers, clinicians, and coaches need to better communicate with and educate parents on the risks of sport specialization so that they can make informed decisions.

Parental desire for sport advancement and college scholarships did not show notable differences between specialized and non-specialized groups. This disagrees with previous literature reporting that 93% of youth baseball parents and over 95% of youth basketball parents believed year-round focus on one sport increased the chances of playing in high school and college, with 99% of baseball parents believing it would improve skill development (23, 24). An investigation of over 1,500 NCAA athletes found that more than 80% of participants were classified as “late specializers” meaning that they did not specialize in a single sport until after the age of 15 (28). Furthermore, the majority of Division 1 athletes played more than one sport during their senior year of high school (29). Alternatively, there was a difference in child desire for sport advancement. While the difference of child desire compared to parental desire for sport advancement is not substantial, children may be more motivated to improve their skills than is shown by parents. Further surveying of youth athletes may demonstrate even greater discrepancies between child and parent desires.

Notably, among the potential motivators that were assessed, “Family Support/Involvement” received the greatest percentage of agreement, highlighting the central role that families and parents have in navigating sport specialization. However, no notable differences were observed between degrees of specialization, indicating that regardless of a child's participation level, family members provide influential assistance. Coach Recommendations showed slight differences between specialized and non-specialized groups. There appears to be an effect of age and sport level (school vs. club) on broad multi-activity exposure and dedicated sport success singularity (30, 31). For children of a younger age, sport leaders appear to be less influential on the decision to focus on a single sport or participate in multiple. Track and field coaches indicated that they believed specialization should occur at a later age than was reported by parents of track and field youth athletes (31). Another study reported that club sport coaches were more likely to view specialization positively, as compared to high school coaches (30). Leaders in a child's athletic life, like parents and coaches, may have an impact on sport decisions and the push for success. The inclusion of parents of older children in the present study may have resulted in more pronounced outcomes related to Coach and Personal Trainer Recommendations. As athletes grow older and experience increases in competition level, the idea of specialization and whether the child is playing the sport for fun or for their future becomes ever more pertinent to the individual and their stakeholders alike.

### 4.1 Limitations

It is important to note that parents responded to the question asking if their child was specialized with either *Yes* or *No* after being presented with a pictorial definition (2), while researcher-determined specialization status was broken into no, low, moderate, and high levels. Previous research has focused on specialization levels which often involve categorizing those without any specialization into the low category. The present study aimed to make comparisons between the parents of specialized and non-specialized athletes by separating *No*'s from *Low*'s, which may have influenced some of our results. Using the three-question scale to determine specialization level raises the issue of whether low specialization should be considered “specialized”, as determined by any single question, or if only those at moderate and high levels ought to be labeled as “specialized” athletes. A standard questionnaire, and the only one being widely implemented at the time, was used to evaluate specialization. A more comprehensive specialization index may provide objective characteristics and classification for future research (26, 32). The present population also exhibited a much smaller percentage of children who were moderate or highly specialized than recorded in previous literature (16, 23, 24, 33). However, the difference in specialization levels and younger age group of the present population may explain some of these differences. Additionally, the age of specialization can vary between sports, such as gymnastics for instance, where athletes typically peak in performance at a much younger age than other sports. Future analyses should investigate how specialization is affected by the sport(s) participants are involved in.

As with any survey, responses represent subjective data and may be affected by recall bias or variable interpretations of questions. The majority of parent participants in this study were white females, indicating potential demographic clustering, and therefore, the results presented here may not be generalizable to all parent populations. Additionally, convenience sampling and snowball recruitment may over-represent specialized participants and certain motivators. Parents were encouraged to complete the survey for all active children in their household, potentially skewing demographics and results. However, it was deemed important to include all children in a household active in sport as they likely differ in age and sport characteristics, and this increased the overall sample size. Certain demographic variables like education and household income were fairly stratified and the children in this study were involved in a wide variety of sports, both team and individual. Even so, additional investigation into the motivation behind sport specialization with a more diverse parent cohort is warranted. Subanalyses into household income and education were reserved for future analyses and therefore, not presented in the current manuscript. Although corrections were applied for *post hoc* comparisons, the evaluation of multiple motivators may increase the risk of Type I error; therefore, findings should be interpreted cautiously and validated in future confirmatory studies. Finally, the survey was developed amongst a group of specialization experts and included a list of potential motivators from sports professionals. However, no formal validation, pilot testing, cognitive interviewing, or psychometric validation with parents was performed. Furthermore, coaches were not included in the survey creation study team in order to form a non-bias group of professionals as coaches may at times have an influence in the decision to specialize. Future work should incorporate all professionals as they would have different insight into the specialization decision-making process.

### 4.2 Conclusions

While Family Support/Involvement and Parent Enjoys Social Opportunity garnered the most agreement of all motivators, it did not differ across specialization status or level. However, children's specialization was commonly associated with lack of time from parents and children alike, as well as the push for sport advancement. Despite the known risks associated with sport specialization, the number of specialized youth athletes continues to rise, garnering necessary insight into the reasoning behind that decision. Importantly, continued parent education on youth specialization is warranted. Seminars, interactive sessions, and one-on-one discussions with health professionals may help bridge the gap in perception and reality of sport specialization. Additionally, time showed to play a critical role in specialization, suggesting that schedules may be too busy to allow for multisport play. Recreational leagues and club leagues often come with vastly different time commitments. Therefore, educating coaches and the leadership of sports organizations on appropriate sport volumes, or adjustment of schedules to allow for participation in multiple sports, could result in valuable outcomes.

## Data Availability

The raw data supporting the conclusions of this article will be made available by the authors, without undue reservation.
