# Supplementary material for: Parental perspectives on youth sport specialization: insights on motivators across specialization levels
Source: Front Sports Act Living. 2026 Feb 10;8:1736749. doi: 10.3389/fspor.2026.1736749 (PMC12929495; doi:10.3389/fspor.2026.1736749)
Supplement: Supplementary file 1 [file Table1.docx]

**APPENDIX A**

| Parents were asked to *please rate the extent to which you agree with the following statements* | |
| --- | --- |
| **Motivator** | **Statement** |
| Parent Lacks Time | I do not have enough time for my child to participate in more than one sport |
| Child Lacks Time | My child does not have enough time to participate in more than one sport due to other extracurricular and scholastic activities |
| Financial Constraints | Our family has financial constraints that limit the amount and type of sport participation |
| Community Lacks Accessibility | Our family lives in an area that has limited access to a variety of sports/activities |
| Team / League Rules | Our team or league does not allow my child to participate in other sports |
| Coach Recommendations | My child’s coach has made recommendations regarding sport participation and/or additional training opportunities outside of my child’s primary sport |
| Parent Desire for College Scholarship | I wish for my child to attain an athletic college scholarship |
| Improve College Application | My child’s sport participation will make their college application more well-rounded |
| Parent Enjoys Social Opportunity | I enjoy the social opportunities and relationships with other parents from my child’s team/sport |
| Parent Enjoys Sport Connection | I enjoy staying connected to a sport that I played in the past |
| Parent Has Made New Friends | My child’s sports participation has allowed me to make new friends |
| Parent Enjoys Parent Interaction | I enjoy interacting with the other parents on my child’s team |
| Parent Would Miss Parent Interaction | If my child stopped participating in their sport, I would miss the interactions with other parents |
| Family Support / Involvement | I like that our entire family can support my child’s sport participation |
| Parent Fear of Injury | I am afraid that my child may become injured if they were to increase their current sport participation/training volume or participate in certain sports |
| Parent Desire for Sport Advancement | I want my child to compete at a higher level, such as in college or professional sports |
| Conflicts with Child Socialization | I do not want my child to miss out on social opportunities with peers due to sports commitments |
| Conflicts with Sport Advancement | I do not want my child to miss out on opportunities to advance in their sport |
| Child Desire for Sport Advancement | My child has shared a desire to play in college or professionally |
| Child Lost Interest in Other Sports | My child lost interest in some sports over others |
| Child Lacks Athletic Ability | My child’s athletic ability does not permit them to participate in a variety of sports |
| Child’s Injury History | My child’s past sport-related injuries have influenced the sports I allow my child to participate in |
| Personal Trainer Recommendations | My child’s personal trainer / fitness coach has made recommendations regarding participation in additional sports and/or training opportunities outside of my child’s primary sport |

Note: Likert-Scale responses ranged from 1 = strongly agree to 5 = strongly disagree
